# Essential Oils: New Perspectives in Human Health and Wellness

**DOI:** 10.1155/2014/467363

**Published:** 2014-06-22

**Authors:** Fabio Firenzuoli, Vikas Jaitak, Gyorgyi Horvath, Imaël Henri Nestor Bassolé, William N. Setzer, Luigi Gori

**Affiliations:** ^1^Center for Integrative Medicine, Referring Center for Phytotherapy, Tuscany Region, Careggi University Hospital, School of Medicine of Florence, 50139 Florence, Italy; ^2^Centre for Chemical and Pharmaceutical Sciences, Central University of Punjab Bathinda, Punjab 151001, India; ^3^Institute of Pharmacognosy, Medical School, University of Pécs, Pécs 7624, Hungary; ^4^Life and Earth Sciences Training and Research Unit, University of Ouagadougou, Ouagadougou, Burkina Faso; ^5^Department of Chemistry, University of Alabama in Huntsville, Huntsville, AL 35899, USA

Essential oils are natural products, derived from aromatic plants, traditionally used all over the world for disinfection, as anti-inflammatory, relaxing, and stimulating substances, and with potential and modern exploitation in clinical medicine. The earliest recorded mention of the techniques and methods used to produce essential oils is believed to be that of Ibn al-Baitar (1188–1248). The chemical composition of these essential oils varies widely depending upon the geographical location, botanical origin, genetics, bacterial endophytes, and extraction techniques. Essential oils are commonly used in food and cosmetic industries. They can be used as natural alternatives to synthetic preparations to prevent and treat infectious diseases. They are used traditionally to treat other diseases like respiratory tract, digestive system, gynecological, andrological, endocrine, cardiovascular, nervous system, and skin infections. Many of them have shown anticancer activities, too.

For this special issue we received seventeen papers, confirming the interest shown by the scientific community on a great number of old and new issues still open about essential oil, particularly chemical characterization and standard methods to control quality; human clinical and experimental pharmacology and toxicology of essential oils; safety; pharmacological actions and interactions; new biomedical targets of the biological responses; clinical assays to pharmaceutical products with essential oils in biotechnology; nanotechnology and nanomedicine. We have selected seven papers, with the aim of showing some progress made by the pharmaceutical point of view and the new perspectives of pharmacological research to facilitate their transition into clinical practice.

In the study* “Hepatoprotective effect of pretreatment with Thymus vulgaris essential oil in experimental model of acetaminophen-induced injury,”* R. Grespan et al. investigated the hepatoprotective effect of* Thymus vulgaris* essential oil on acetaminophen-induced hepatic damage in mice.* Thymus vulgaris* is used popularly such as its antiseptic, carminative, and antimicrobial effects, and this study is very interesting for new possible clinical applications. The positive results are confirmed by reduction of the serum marker enzymes aspartate aminotransferase (AST), alanine aminotransferase (ALT), and myeloperoxidase (MPO) and by histopathological analysis. Acute liver damage caused by acetaminophen overdose is a significant clinical problem and could benefit from new therapeutic strategies.

In the paper entitled* “Exploring the anti-Burkholderia cepacia complex activity of essential oils: a preliminary analysis,”* I. Maida et al. have checked the ability of the essential oils extracted from six different aromatic plants to inhibit the growth of 18 known species of the* Burkholderia cepacia* complex (Bcc), an opportunistic human pathogen that can cause severe infection in immunocompromised patients, especially those affected by cystic fibrosis (CF) and who are often resistant to multiple antibiotics.* Eugenia caryophyllata*,* Origanum vulgare,* and* Thymus vulgaris* essential oils were particularly active versus the Bcc strains, including those exhibiting a high degree or resistance to ciprofloxacin, active toward both environmental and clinical strains isolated from CF patients.

N. El Abed et al. in the work* “Chemical composition, antioxidant and antimicrobial activities of Thymus capitata essential oil with its preservative effect against Listeria monocytogenes inoculated in minced beef meat”* evaluated the preservative effect of* Thymus capitata* essential oil (TCEO) against* Listeria monocytogenes* inoculated in minced beef meat. The antioxidant activity was assessed in vitro by using both the DPPH and the ABTS assays. The essential oil was evaluated for its antimicrobial activity using disc agar diffusion and microdilution methods. The results demonstrated that the minimum inhibition concentration values ranged from 0.32 to 20 mg/mL, and essential oil evaluated in vivo against* Listeria monocytogenes* showed clear and strong inhibitory effect. The application of 0.25 or 1% (v/w) of TCEO to minced beef significantly reduced the* L. monocytogenes* population.

In the paper* “Essential oils for complementary treatment of surgical patients: state of the art”* S. Stea et al. revised the available literature to determine the effectiveness of aromatherapy in surgical patients (to treat anxiety and insomnia, pain and nausea, or to dress wounds). Efficacy studies of lavender or orange and peppermint essential oils, to treat anxiety and nausea, respectively, have shown positive results. Therefore there are encouraging data for the treatment of infections, especially for tea tree oil (TTO). The authors conclude that it is important that the therapeutic use of essential oils be performed in compliance with clinical safety standards.

In the article* “Effect of eugenol on cell surface hydrophobicity, adhesion, and biofilm of Candida tropicalis and Candida dubliniensis isolated from oral cavity of HIV-infected patients”* S. B. de Paula et al. evaluated the effect of eugenol on the adherence properties and biofilm formation capacity of* Candida dubliniensis* and* Candida tropicalis* isolated from the oral cavity of HIV-infected patients. Eugenol showed inhibitory activity against planktonic and sessile cells of* Candida* spp. Scanning electron microscopy demonstrated that eugenol drastically reduced the number of sessile cells on denture material surfaces. The paper corroborates the effectiveness of eugenol against* Candida* species other than* C. albicans*, reinforcing its potential as an antifungal applied to limit both the growth of planktonic cells and biofilm formation on different surfaces.

In the work* “Essential oils loaded in nanosystems: a developing strategy for a successful therapeutic approach”* A. R. Bilia et al. revised the nanoencapsulation of essential oils in drug delivery systems, for their capability of decreasing volatility, improving the stability, water solubility, and efficacy of essential oil-based formulations, by maintenance of therapeutic efficacy. Two categories of nanocarriers can be proposed: polymeric nanoparticulate formulations, extensively studied with significant improvement of the essential oil antimicrobial activity, and lipid carriers, including liposomes, solid lipid nanoparticles, nanostructured lipid particles, and nano/microemulsions.

Lastly, the paper by A. R. Bilia et al. entitled* “Essential oil of Artemisia annua *L.*: an extraordinary component with numerous antimicrobial properties”* describes the qualitative composition and the antimicrobial activities of essential oil of* Artemisia annua* L., a medicinal plant from China, well known and used in the treatment of the chloroquine-resistant and cerebral malaria. The essential oil of* A. annua* is rich in mono- and sesquiterpenes (camphor, germacrene D, artemisia ketone, and 1,8 cineole) exciting a lot of antibacterial and antifungal activities: against gram-positive bacteria (*Enterococcus, Streptococcus, Staphylococcus, Bacillus*, and* Listeria* spp.), gram-negative bacteria (*Escherichia, Shigella, Salmonella, Haemophilus, Klebsiella*, and* Pseudomonas* spp.), and mycetes (*Candida, Saccharomyces*, and* Aspergillus* spp.). The authors believe that this review will serve to prepare the most appropriate microbiological studies also useful for clinical practice.

*Fabio Firenzuoli* *Fabio Firenzuoli* *Vikas Jaitak* *Vikas Jaitak* *Gyorgyi Horvath* *Gyorgyi Horvath* *Imaël Henri Nestor Bassolé* *Imaël Henri Nestor Bassolé* *William N. Setzer* *William N. Setzer* *Luigi Gori* *Luigi Gori*

